# 2D Phase Formation on 3D Perovskite: Insights from Molecular Stiffness

**DOI:** 10.1021/acsami.4c11394

**Published:** 2024-09-13

**Authors:** Lucas Scalon, Charles Alves Nogueira, André Felipe Vale Fonseca, Paulo E. Marchezi, Raphael Fernando Moral, Giulia Grancini, Tim Kodalle, Carolin M. Sutter-Fella, Caio Costa Oliveira, Luiz F. Zagonel, Ana F. Nogueira

**Affiliations:** † Institute of Chemistry, University of Campinas (UNICAMP), 13083-970 Campinas, São Paulo, Brazil; ‡ Gleb Wataghin Institute of Physics, University of Campinas (UNICAMP), 13083-859 Campinas, São Paulo, Brazil; § Department of Nanoengineering, UC San Diego, 9500 Gilman Drive, La Jolla, California 92093, United States; ∥ Molecular Foundry, Lawrence Berkeley National Laboratory, 1 Cyclotron Road, Berkeley, California 94720, United States; ⊥ Department of Chemistry and INSTM, University of Pavia, Via T. Taramelly 14, 27100 Pavia, Italy; # Advanced Light Source, Lawrence Berkeley National Laboratory, 1 Cyclotron Road, Berkeley, California 94720, United States

**Keywords:** 2D/3D perovskites, low-dimensional perovskites, perovskite solar cells, in situ GIWAXS, SEM-cathodoluminescence

## Abstract

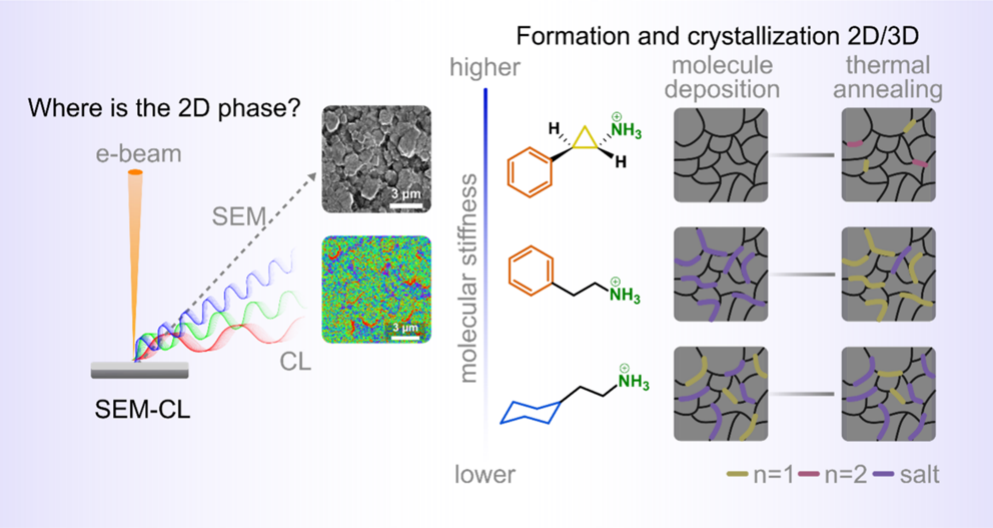

Several studies have demonstrated that low-dimensional structures (*e.g*., two-dimensional (2D)) associated with three-dimensional (3D) perovskite films enhance the efficiency and stability of perovskite solar cells. Here, we aim to track the formation sites of the 2D phase on top of the 3D perovskite and to establish correlations between molecular stiffness and steric hindrance of the organic cations and their influence on the formation and crystallization of 2D/3D. Using cathodoluminescence combined with a scanning electron microscopy technique, we verified that the formation of the 2D phase occurs preferentially on the grain boundaries of the 3D perovskite. This helps explain some passivation mechanisms conferred by the 2D phase on 3D perovskite films. Furthermore, by employing *in situ* grazing-incidence wide-angle X-ray scattering, we monitored the formation and crystallization of the 2D/3D perovskite using three cations with varying molecular stiffness. In this series of molecules, the formation and crystallization of the 2D phase are found to be dependent on both steric hindrance around the ammonium group and molecular stiffness. Finally, we employed a 2D/3D perovskite heterointerface in a solar cell. The presence of the 2D phase, particularly those formed from flexible cations, resulted in a maximum power conversion efficiency of 21.5%. This study provides insight into critical aspects related to how bulky organic cations’ stiffness and steric hindrance influence the formation, crystallization, and distribution of 2D perovskite phases.

## 1 Introduction

Hybrid organic–inorganic perovskites are promising materials for application as light absorbers in solar cells. In-lab results have demonstrated that perovskite solar cells (PSCs) can achieve power conversion efficiency (PCE) as high as 26%, which is comparable to that of crystalline silicon-based solar cells.^1^ One of the key aspects of PSC’s high efficiency is the interface passivation of the perovskite, which is essential due to the high density of charge carrier trap states typically observed across the interfaces.^2−4^ The interface passivation, besides decreasing the density of trap states, is reported to improve perovskite stability against moisture degradation,^5,6^ decrease hysteresis,^7,8^ and suppress ion migration.^9−11^

Different types of compounds can be employed to passivate the three-dimensional (3D) perovskite surface, including inorganic salts,^12−14^ polymers,^15^ oxides,^16−18^ graphene,^19−21^ two-dimensional (2D) carbides,^22,23^ and organic molecules.^5,24,25^ Among these, organic molecules are of particular interest.^6^ They can be designed with specific organic groups to passivate surface trap states through Lewis acid–base interactions.^26^ A common organic functional group is the ammonium (−NH_3_^+^) group, which can be attached to an aliphatic chain or aromatic unit.^27^ Frequently, ammonium-based organic molecules feature a central core such as an aryl or cycloalkyl ring and an alkylammonium chain. Example of molecules include phenylethylammonium and thiophenemethylammonium. The ammonium group on these molecules can react with the 3D perovskites, leading to the formation of low-dimensional phases. This reaction occurs via a substitution mechanism, whereby the bulky ammonium-based organic cation replaces the small cation (such as Cs^+^, MA^+^, and/or FA^+^) on the surface of the film. The resulting product is a 2D perovskite with the chemical formula (A’)_2_(A)_*n-*1_Pb_*n*_X_3*n*+1_, in which A’ is the bulky organic cation, A is the small-size cation, and X is a halide (Cl^–^, Br^–^, I^–^). The integer *n* determines the thickness of the inorganic layers of the perovskite, thereby defining the quantum confinement regime of the 2D phase. The formation of 2D/3D perovskite heterointerfaces has been demonstrated to enhance the efficiency and stability of PSC.^28−30^

**Figure 1:**
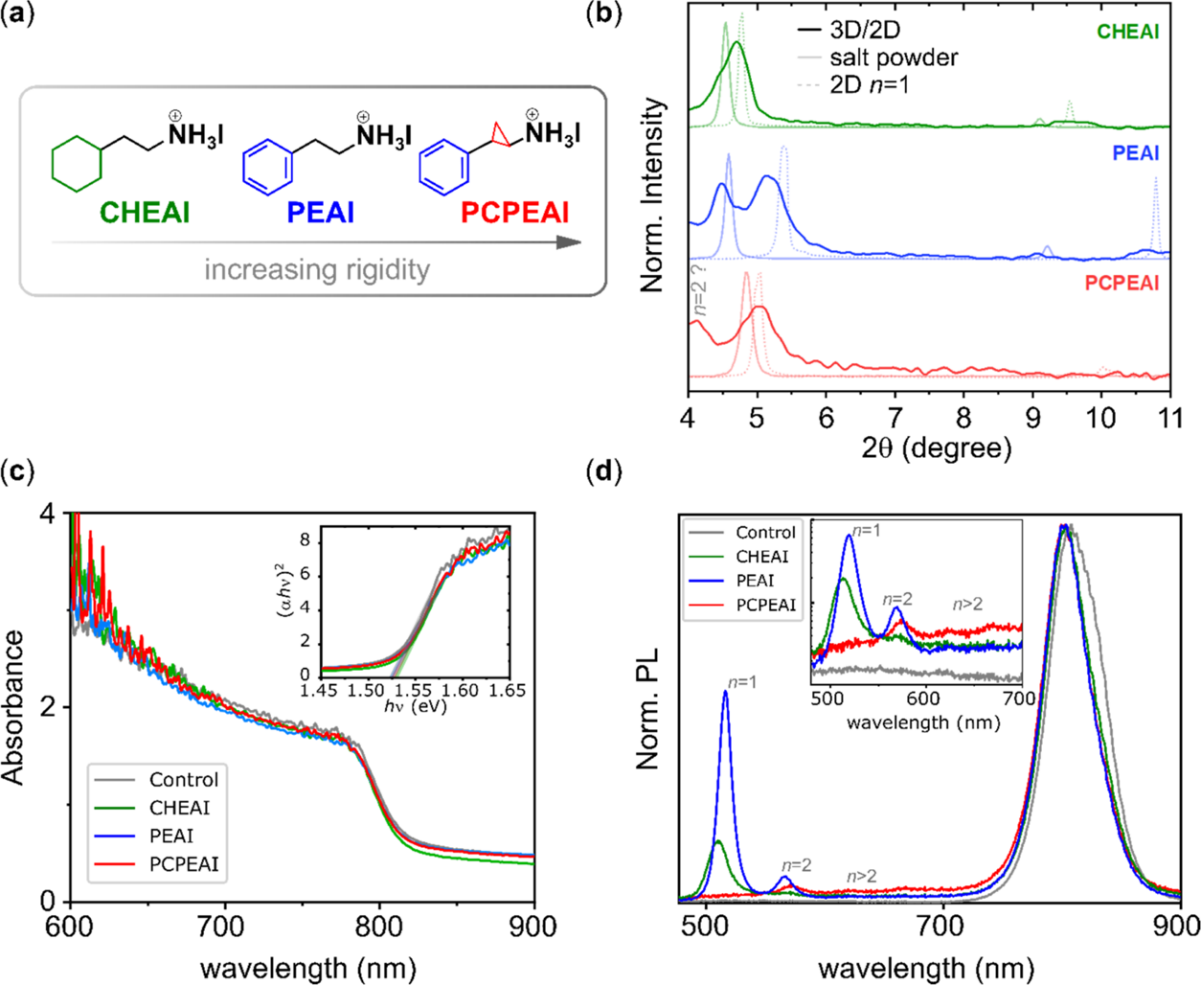
(a) Chemical structures of the organic cations studied in this work. (b) X-ray diffraction of the 2D/3D perovskite (bold lines), salt powder (shadow lines), and pure 2D *n* = 1 perovskite phase (dotted line). (c) UV–vis and (d) steady-state PL spectra of the 2D/3D perovskite. The insets in (c) and (d) are the Tauc plot of the absorption edge and the zoom-in in the region of low-dimensional phase emission, respectively. All measurements were performed in the 2D/3D perovskite films after thermal annealing at 100 °C for 10 min.

In this work, we seek to elucidate the distribution, formation, and crystallization of the 2D/3D heterointerface prepared from cations with varying molecular stiffness and steric hindrance around the ammonium group. To this goal, phenylethylammonium iodide (PEAI), cyclohexylethylammonium iodide (CHEAI), and *trans*-2-phenylcyclopropylammonium iodide (PCPEAI) are compared. The chemical structures of these molecules are shown in Figure 1a. CHEAI is more flexible than PEAI due to the presence of a cyclohexyl ring instead of a phenyl ring. Both molecules feature the same ethylammonium chain. In contrast, PCPEAI, despite sharing the same aryl group as PEAI, features an ammonium group attached to a cyclopropyl chain. This feature results in both an increase in the molecular stiffness and the steric hindrance of the ammonium group.

First, we employed cathodoluminescence combined with scanning electron microscopy (SEM-CL) to track the distribution of the 2D phases onto the 3D perovskite. The results demonstrate that the 2D perovskite is primarily located at the grain boundaries of the 3D film. Given that the grain boundaries act as nonradiative recombination centers,^31,32^ the presence of the 2D phase at these locations helps elucidate the enhanced photovoltaic performance observed when adopting 2D/3D heterointerfaces. Having established the location of the 2D phase on the 3D perovskite film, we monitor the formation and crystallization dynamics of the 2D/3D heterointerface constructed from the three different cations using*in situ* grazing-incidence wide-angle X-ray (GIWAXS) technique. This technique has been shown to be a very effective tool at revealing transient nucleation and crystallization stages and processes in 2D^33,34^ and 3D perovskites.^25,35^ GIWAXS results indicate that the formation and crystallization of the 2D phase are influenced by both the steric hindrance on the ammonium group and the stiffness of the molecule. The incorporation of the 2D/3D heterointerfaces into solar cells is observed to result in enhanced efficiency gains when the organic cation is flexible and less sterically hindered.

## 2 Experimental Section

### 2.1 Synthesis of Phenylethylammonium Iodide (PEAI)

For the PEAI, 1.040 mL (8.25 mmol) of phenylethylamine was added to a round-bottom flask with 2 mL of ethanol. The solution was cooled in an ice bath and stirred for 20 min. Then, 2 mL (1.1 equiv, 9.08 mmol) of HI solution 57 wt % was added dropwise into the reaction. After 2 h, the solvent was evaporated, and the solid residue was dissolved in a minimal amount of hot ethanol. This solution was allowed to rest in the refrigerator overnight, and white crystals were formed. The crystals were then filtered, washed several times with diethyl ether, and dried under vacuum. This procedure was repeated three times to ensure the removal of all HI residue.

### 2.2 Synthesis of Cyclohexylethylammonium Iodide (CHEAI)

For CHEAI, the synthesis protocol was the same as that for PEAI, except that the precursor used was cyclohexylethylamine.

### 2.3 Synthesis of *trans*-2-Phenylcyclopropylammonium Iodide (PCPEAI)

For PCPEAI, we used *trans*-2-phenylcyclopropylammonium chloride (PCPEACl) as a precursor. The replacement of chloride by iodide counteranion is not thermodynamically favored; therefore, an additional step to deprotonate the PCPEACl and obtain *trans*-2-phenylcyclopropylamine is required. To do so, 500 mg of PCPEACl was dissolved in 5 mL of dichloromethane and transferred to a separatory funnel. Then, 5 mL of 1 M NaOH in water was added to the funnel and mixed vigorously. The organic phase was collected, and this procedure was repeated three times. The organic phases were combined and dried under reduced pressure, obtaining a yellow oil of *trans*-2-phenylcyclopropylamine. This oil was then used to prepare PCPEAI following the protocol used for PEAI and CHEAI.

### 2.4 Synthesis of Phase-Pure *n* = 1 2D (CHEA, PEA, PCPEA)_2_PbI_4_ Perovskite Thin Films

To a vial, 3 mmol of PbI_2_ and 6 mmol of the corresponding iodide-based salt, either CHEAI, PEAI, or PCPEAI, were dissolved with 1 mL of *N*,*N*-dimethylformamide (DMF). The solution was stirred for 30 min at room temperature. Thin films of these perovskites were prepared by spin coating the precursor solution at 1000 rpm for 10 s with 500 rpm s^–1^ of acceleration, followed by 4000 rpm for 30 s, with 1000 rpm s^–1^ of acceleration. Finally, the film was thermally annealed at 100 °C for 10 min.

### 2.5 Precursor Solution Preparation

#### 2.5.1 Cs_0.05_(FA_0.83_MA_0.17_)_0.95_PbI_3_ (CsFAMA)

The protocol was adapted from refs (36,37). For the preparation of Cs_0.05_(FA_0.83_MA_0.17_)_0.95_PbI_3_, a 1.5 M solution of CsI in dimethyl sulfoxide (DMSO) and a 2.5 M solution of PbI_2_ in DMF/DMSO (4:1) were prepared by heating them at 140 °C. Next, the density of each solution was determined, and the true concentration was calculated. The true concentration is lower than the target concentration due to the expansion of the solution volume. Then, the CsI was added to the PbI_2_ solution in a 0.05:0.95 mol/mol ratio, forming an inorganic perovskite solution of Cs_0.05_PbI_3_, which was stirred at room temperature for 30 min. In two separate vials, FAI and MAI were weighed, and the inorganic perovskite solution was added in a 0.95:1 molar ratio, resulting in Cs_0.05_FA_0.95_PbI_3_ and Cs_0.05_MA_0.95_PbI_3_ perovskites; each solution was stirred at room temperature for 30 min. Next, the MA-containing perovskite was added to the FA perovskite in a 5:1 (v/v) ratio, obtaining a 1.418 M Cs_0.05_(FA_0.83_MA_0.17_)_0.95_PbI_3_ solution that was stirred at room temperature for 1 h. To this solution, 5% of PbI_2_ (in relation to the amount of Pb) was added from a 2.5 M solution of PbI_2_ in DMF/DMSO 4:1 v/v. After the addition of the excess PbI_2_, the perovskite solution concentration decreases to 1.35 M. The perovskite solution was filtered before deposition using a 0.45 μm PFTE filter and deposited by spin coating at 1000 rpm for 10 s, with 1000 rpm s^–1^ of acceleration, followed by 5000 rpm for 30 s, with 1000 rpm s^–1^ of acceleration. In the final 10 s of the spin-coating program, 200 μL of chlorobenzene was quickly added to the perovskite film using a 1 mL pipet tip, keeping a distance pipet tip–substrate of ∼1 cm. We noticed that the slow addition of the antisolvent resulted in cracks on the perovskite film surface and bad adherence of the film to the SnO_2_ layer, both worst solar cell performance. The films were then annealed at 100 °C for 30 min.

#### 2.5.2 Cs_0.10_FA_0.90_PbI(I_0.90_Br_0.10_)_3_ (CsFA)

To a vial 28.4 mg of CsBr, 646.0 mg of PbI_2_ and 206.5 mg of FAI were mixed in 1 mL of DMF/DMSO (4:1). The solution was stirred at room temperature for 1 h. Then, 14.2 mg of MACl was added directly into the perovskite precursor, and the solution was stirred for more than 1 h. In sequence, the solution was filtered with a 0.45 μm PFTE filter and deposited onto the FTO/SnO_2_ substrate at 4000 rpm for 30 s, with 1000 rpm s^–1^ of acceleration. In the final 10 s of the spin-coating program, 200 μL of chlorobenzene/2-propanol (9:1) was quickly added to the perovskite film using a 1 mL pipet tip, keeping a distance pipet tip–substrate of ∼1 cm. After spin coating, the films were thermally annealed at 100 °C for 3 min and removed from the hot plate. Final annealing at 120 °C for 30 min was performed inside the glovebox to complete the perovskite crystallization and remove the excess of MACl.

## 3 Results

### 3.1 Impact of the Molecular Flexibility on the Formation of the Low-Dimensional Perovskite Phase

A perovskite composition based on Cs_0.05_(FA_0.87_MA_0.13_)_0.95_PbI_3_ (hereafter abbreviated as CsFAMA) was adopted. A slight excess of lead iodide (5 mol %) was employed due to its ability to reduce the halide vacancy concentration^38^ and increase the perovskite stability.^39^ The 2D phase was formed *in situ* onto the 3D film by dynamically depositing a 40 mM solution of the CHEAI, PEAI, and PCPEAI salts in 2-propanol, followed by a thermal annealing step at 100 °C for 10 min. Further details about sample preparation can be found in the Experimental Section and the Support Information (SI).

The X-ray diffraction (XRD) pattern (Figure S1) of the 2D/3D films shows a diffraction corresponding to the (001) plane of the 3D perovskite at ∼13.8°. Additionally, a peak corresponding to PbI_2_ is observed at 12.5°. The addition of the bulky organic cation results in a decrease in the relative intensity of the PbI_2_ peak and the emergence of new diffraction features at the low angle region (2θ < 7°), which are indicative of the formation of 2D perovskites (Figure S1). Figure 1b shows an enlarged view of the low-angle region of the diffractogram of the 2D/3D perovskites, the phase-pure *n* = 1 2D perovskites, and the pristine salts.^40^ A comparison of the plots reveals that 2D/3D perovskites exhibit diffraction peaks corresponding to the salt and the *n* = 1 2D perovskite.^25,41,42^ For PCPEAI treatment, an additional peak at about 4.3° can also be observed, which is probably related to the *n* = 2 (020) plane of 2D perovskite.^40,43,44^

The surface morphology of the 2D/3D perovskite films was monitored by using scanning electron microscopy (SEM). As depicted in Figure S2, SEM images show a heterogeneous topography of the treated films compared to the pristine 3D perovskite (Figure S2a). While CHEAI and PCPEAI show sharp needle-like features of the organic salts and 2D perovskites (in agreement with XRD of Figure 1b), the PEAI shows opaque platelike morphology on the surface. In all cases, these features are caused by the formation of 2D perovskites and some deposition of unreacted salts. These features hinder the visualization of the grains of the underlying 3D perovskite. In these images, a color contrast between darker and brighter regions suggests the existence of amorphous and crystalline domains, respectively, or wrinkles in the film.

To gain further insight into the 2D phases prepared from cations with varying molecular stiffness, we performed XRD measurements of pure *n* = 1 phases. Figure S3 compares the XRD of the pure *n* = 1 phases with the diffraction of the corresponding iodide-based salt. The interlayer spacing (*d*, as determined by Bragg’s law) between the lead octahedra sheets decreases in the order CHEAI > PCPEAI > PEAI, from 18.6 to 17.5 and 16.3 Å, respectively. The cation CHEA^+^ is the most flexible of the three, presenting a cyclohexane ring in a chair conformation instead of a benzene ring in a flat conformation as in PEA^+^ and PCPEA^+^. The greater interplanar distance is, therefore, a result of the presence of additional hydrogen atoms and increased motion and mobility degrees of freedom of the cyclohexyl ring. Additionally, CHEAI lacks π–π stacking interactions observed in PEAI and PCPEAI, which contributes to an increased average separation distance between the molecules. Conversely, although PCPEA^+^ shares the same phenyl group as PEA^+^, it is constrained by a configurational restriction caused by the cyclopropane group in the alkylammonium chain. The presence of the cyclopropane group limits the degree of rotational freedom of the alkyl chain, which increases the average intermolecular distance. This results in a decreased π–π stacking interaction when compared to PEAI. Consequently, the organic cage of the *n* = 1 (PCPEA)_2_PbI_4_ 2D perovskite is larger than that of (PEA)_2_PbI_4_.

The UV–vis absorption and steady-state photoluminescence (SSPL) spectra of the 2D/3D and pristine 3D perovskite were also recorded. The 3D perovskite has a band gap energy of 1.53 eV, as estimated from the inset Tauc plots (Figure 1c), and an emission peak at 800 nm (Figure 1d). The formation of the 2D phases can be observed in the high-energy region of the emission spectra (inset of Figure 1d). For CHEAI and PEAI, emissions corresponding to the *n* = 1 and *n* ≥ 2 are observed.^25,45^ However, in the case of PCPEAI, no emission corresponding to the *n* = 1 phase can be identified. Given that the *n* = 1 phase has been identified in the XRD of the PCPEAI-based 2D/3D perovskite, it is reasonable to conclude that this phase exhibits a low photoluminescence quantum yield, which precludes its discernibility in the SSPL spectrum.

### 3.2 Spatial Distribution of the Low-Dimensional Perovskite Phases

Our previous results have demonstrated the formation of low-dimensional phases using the CHEAI, PEAI, and PCPEAI cations. However, spatial resolution was insufficient to ascertain their location on the film. An understanding of the location of the 2D phase is pertinent to a more comprehensive understanding of its passivation mechanism. To ascertain the location of the preferential formation of the 2D phase and to monitor the potential impact on molecular stiffness and steric hindrance, we conducted cathodoluminescence (CL) in an SEM. A description of the SEM-CL technique is provided in the SI Note 1.

The SEM-CL technique enables the investigation of the perovskite films at varying depths. This is achieved by varying the electron accelerating voltage, as detailed in SI Note 2. To illustrate, a reduction in the electron accelerating voltage from 5.0 to 1.5 kV results in a decrease in the interaction volume depth in the perovskite from 100 to 30 nm.^46^

We notice that upon formation of the 2D perovskite phase, the characteristics of the 3D grains are no longer discernible (Figure S2). In contrast, large and inhomogeneous features appear. These features are particularly evident when a low accelerating voltage is employed (Figure S5a). Conversely, when the accelerating voltage is increased, the 3D perovskite grains become visible, allowing the location of the grain boundaries (GB) (Figure S5c). It is also important to highlight that the penetration depth of the electron energy is considerably less than the thickness of the film (∼600 nm), and, therefore, the effects of the roughness on the measured CL signal are expected to be minimal.^47^

**Figure 2:**
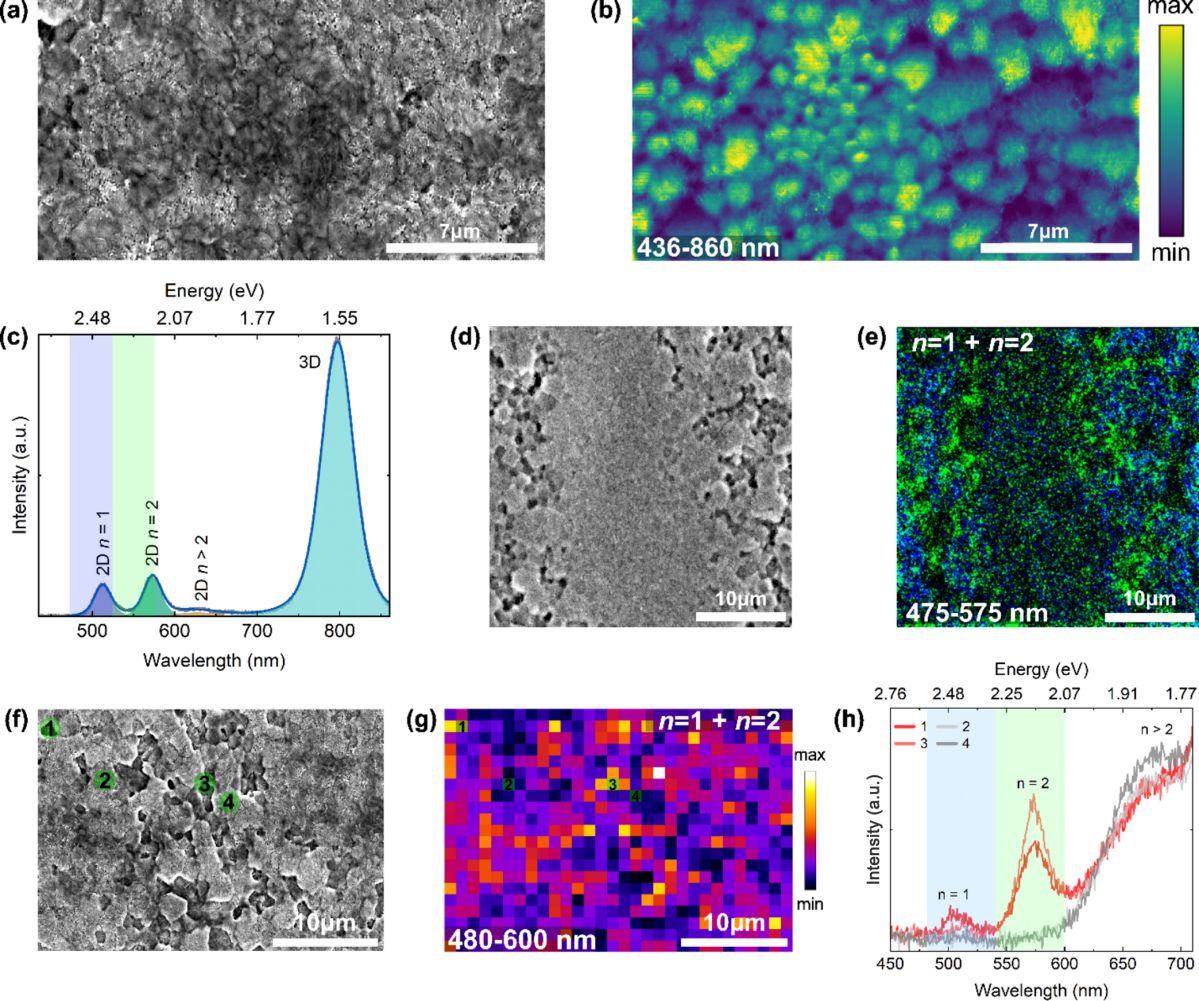
(a) SEM image of the CHEAI-based 2D/3D perovskite acquired simultaneously with the (b) panchromatic image using 5 kV and 50 pA. (c) Large area CL spectra collected for the spectral region of 435–860 nm, acquired with 2 kV and 50 pA. (d) The SEM image at a different region acquired using 10 kV and 100 pA. (e) Overlap of the *n* = 1 and *n* = 2 2D emissions. (f) The SEM image of the CHEAI-based 2D/3D, and (g) the corresponding CL mapping of this region (obtained in the spectrum imaging mode and filtered at 480–600 nm) acquired with 5 kV and 50 pA. (h) Individual CL spectra for pixels 1, 2, 3, and 4, which are indicated in (f) and (g). All of the measurements were performed in 2D/3D perovskite films after thermal annealing at 100 °C for 10 min.

We started by mapping the optical activity in a large region (∼21 μm in width) for the CHEAI-based 2D/3D perovskite. Figure 2a depicts the SEM image of the region of interest in which an accelerating voltage of 5 kV was used to probe the surface of the 2D film. Figure 2b presents the unfiltered CL image (also referred to as a panchromatic image), corresponding to the spectral range of 436–860 nm. The luminescence spectrum from this region (Figure 2c) is consistent with the results from SSPL, indicating the presence of *n* = 1 and *n* ≥ 2 2D perovskite as well as the 3D phase. As illustrated in Figure 2b, the optical activity is heterogeneous, displaying inhomogeneous emission intensities with bright regions interspersed with darker ones.

In order to monitor the distribution of a given *n*-value 2D phase within a specific region of the film, a filter was placed before the detector to capture the emission in a well-defined wavelength range (as opposed to integrating all of the luminescence). Figure 2d depicts a region of interest in CHEAI-based 2D/3D perovskite. For this analysis, a nonhomogeneous region was deliberately selected. The luminescence was monitored in two distinct wavelength ranges: 475–525 nm (*n* = 1 phase, blue region) and 525–575 nm (*n* = 2 phase, green region). Figure S6 presents the individual panchromatic images of the two regions, while a superposition of these images is shown in Figure 2e. It is evident that regions exhibiting a lower intensity of the 2D *n* = 1 phase display a bright emission of the 2D *n* = 2 phase. Moreover, some regions exhibit a reduced luminescence intensity for both phases. These results suggest the possibility that certain regions have a higher concentration of a specific 2D phase, indicating that the distributions of the *n* = 1 and *n* = 2 phases may not always be correlated. Our findings are consistent with those previously observed for 2D perovskite crystals.^48,49^ It should be noted that an identical analysis was conducted on PCPEAI-based 2D/3D perovskites (Figure S8). The observed features for this heterointerface are consistent with the results obtained for the other two cations.

To gain further insight into the unequal distribution of the low-dimensional phases, we conducted SEM-CL in a distinct area of the film (Figure 2f,g). Four representative regions were selected, each one corresponding to a pixel size of (900 × 900) nm. The luminescence spectra of these regions are shown in Figure 2h. These images reveal that the emission features corresponding to the low-dimensional perovskites (450–710 nm) are more evident in pixels 1 and 3 than in pixels 2 and 4. This result corroborates our previous hypothesis regarding the uneven distribution of the 2D perovskite across the 3D film.

**Figure 3:**
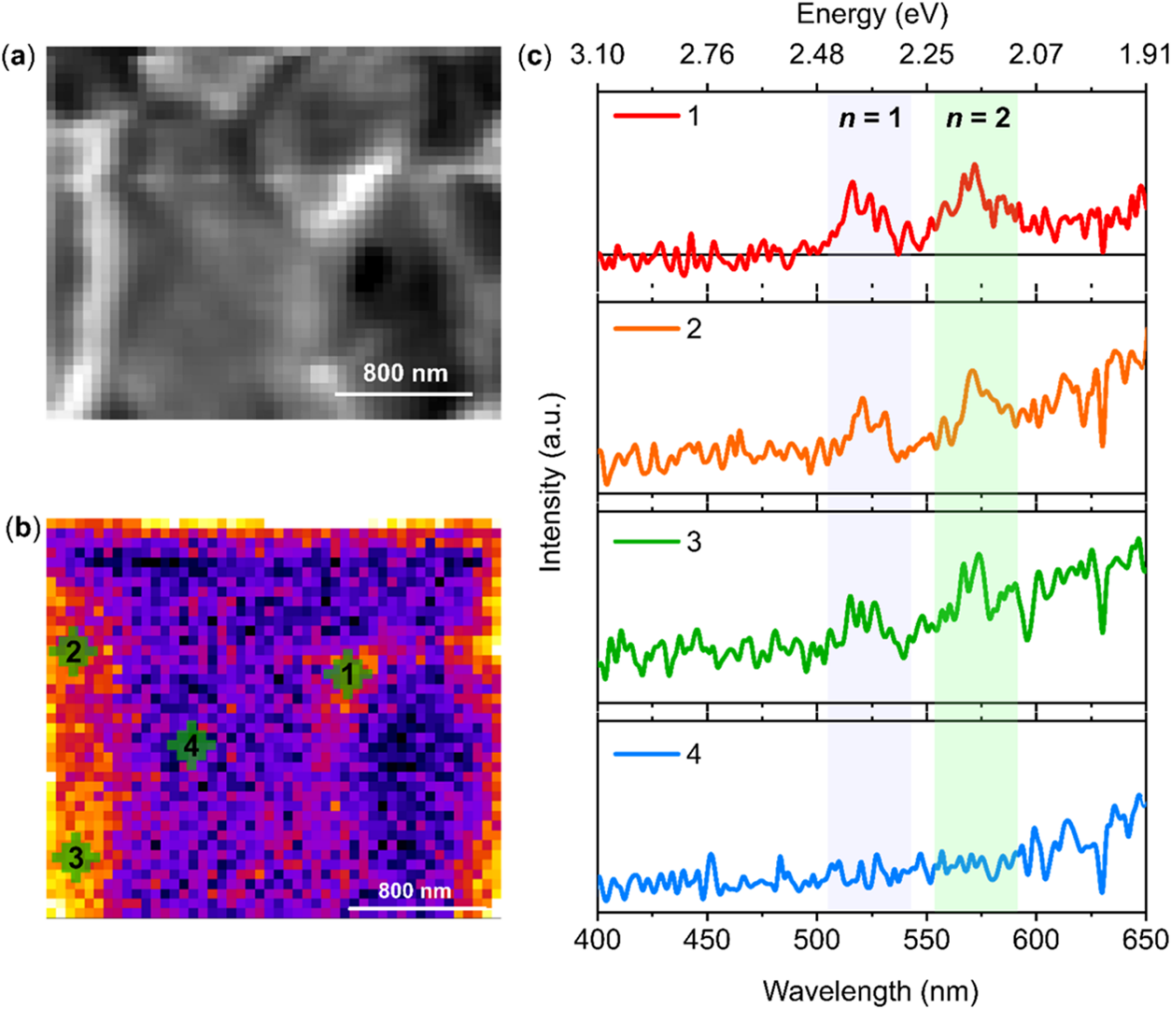
(a) SEM of the PEAI-based 2D/3D perovskite film. (b) CL map of the same region in (a), collected for the spectral region of 400–650 nm, acquired with 5 kV and 25 pA. (c) Individual CL spectra of pixels 1, 2, 3, and 4 assigned in (b). All of the measurements were performed in 2D/3D perovskite film after thermal annealing at 100 °C for 10 min.

We achieved greater magnification and higher spatial resolution in the PEAI-based 2D/3D perovskite and performed CL in the spectral imaging mode. Figure 3a depicts the SEM image of an area in the sample where the GBs of the 3D perovskite can be discerned. The corresponding CL map of this area (Figure 3b) reveals that the 2D phase emission is predominantly concentrated at the GBs. To better demonstrate this, we monitored the luminescence (Figure 3c) of spots 1–4, which are positioned at GBs (spots 1, 2, and 3) or away from the GB (spot 4). Here, each spot corresponds to a pixel size of ∼(40 × 40) nm. As can be observed, the emission intensities of the *n* = 1 and *n* = 2 phases are higher at the GB.

These results, along with the panchromatic CL image (Figure S7), suggest that the 2D phase exhibits a higher intensity at GBs. However, while the 2D phase is present preferentially on the GBs, it is not a universal phenomenon across all GBs. In other words, the distribution of the 2D phase is heterogeneous even across the GBs. This observation suggests two key conclusions: first, the composition of the GB is not uniform across all grains, and second, there are specific GB properties (such as composition, thickness, orientation, etc.) in which the formation of the 2D phase is favored.

Our findings, which span from the micro to nanoscale, provide compelling evidence for the preferential formation of the 2D perovskite at GBs.^29,50,51^ This can be explained by earlier reports indicating that photoluminescence at the grain boundaries is lower than in the interior of the grain.^49,52^ Such a characteristic is associated with the higher trap density of states observed in polycrystalline perovskite.^3,53^ The presence of defects at the grain boundary, including vacancies,^54^ impurities, dangling bonds,^55^ and unreacted PbI_2_ render them inherently a higher reactivity.^56^ Consequently, the formation of the low-dimensional perovskite phases at the GB is facilitated in comparison to that in the grain interior, for instance. As a consequence of the higher trap density of states at the GB, nonradiative recombination of charge carriers occurs at these locations. In a solar cell, this has an effect on the photovoltage of the device, which in turn affects its efficiency. In this regard, the formation of the 2D layer at the GBs inactivates trap centers in the perovskite film, thereby enhancing the power conversion efficiency of the solar cell.

### 3.3 Formation Dynamics of Low-Dimensional Perovskite Phases

*In situ* GIWAXS technique was employed to elucidate the formation and temporal crystallization of the 2D/3D interfaces prepared from CHEAI, PEAI, and PCPEAI. In this experiment, a solution of the cation in 2-propanol was deposited on the CsFAMA perovskite at *t* ∼ 10 s. The spin-coating process was continued for ∼90 s, after which the sample was subjected to thermal annealing at 100 °C for 500 s. The GIWAXS pattern was recorded during the experiment using an incident X-ray angle of 1°. Further details can be found in the SI.

The three molecules studied exhibit differences in terms of stiffness in both the aryl and alkyl constituents. For example, CHEAI exhibits a more flexible structure than PEAI due to the presence of a cyclohexyl ring instead of a phenyl ring. In contrast, PCPEAI features a cyclopropane ammonium chain in place of the linear ethylammonium, which is the case with PEAI. In this regard, while CHEAI and PEAI possess an identical ethylammonium moiety, PCPEAI exhibits a distinct one. The presence of the cyclopropyl substituent in PCPEAI restricts the rotation of the ethylammonium chain in addition to increasing the steric hindrance on the ammonium group. The effects of molecular stiffness on the ammonium chain and aryl substituent can be distinguished within this series of molecules.

**Figure 4:**
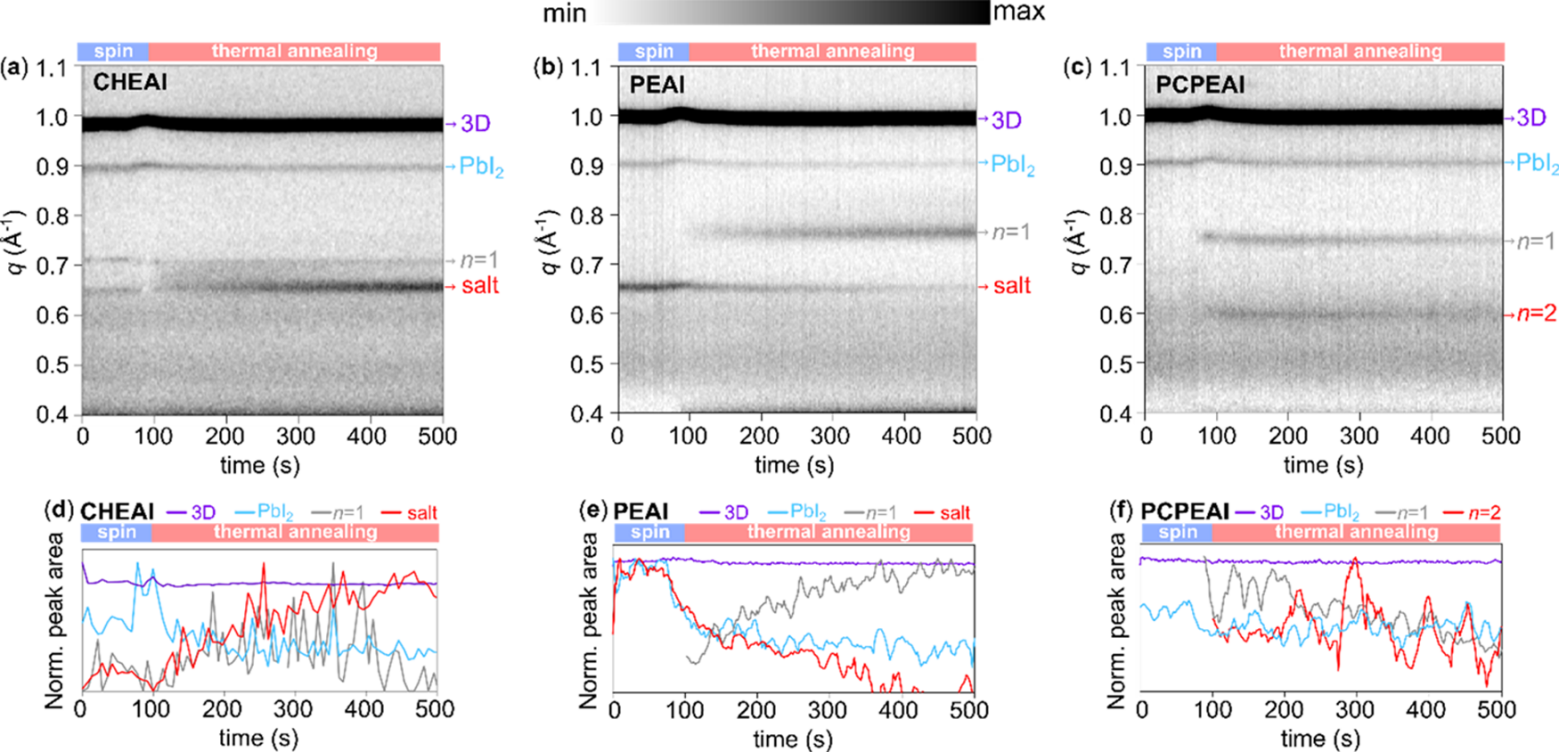
(a–c) In situ GIWAXS color maps as a function of time and (d–f) normalized peak intensity as a function of time of CHEAI, PEAI, and PCPEAI-based 2D/3D perovskites recorded during the spin-coating deposition of the organic cation and thermal annealing at 100 °C. The log_10_ scale was applied to the intensity.

Figure 4a–c presents the *in situ* GIWAXS time evolution of the 2D/3D perovskites prepared from CHEAI, PEAI, and PCPEAI, respectively. The signals at ∼1.0 and 0.87 Å^–1^ are associated with the (001) diffraction plane of the 3D perovskite and PbI_2_, respectively. Furthermore, for CHEAI and PEAI, low *q*-value signals associated with the unreacted salt (∼0.67 Å^–1^) and the *n* = 1 phase of the 2D perovskite (∼0.72 Å^–1^) are observed. For the PCPEAI, both *n* = 1 (at ∼0.72 Å^–1^) and *n* = 2 (at ∼0.60 Å^–1^) phases are present. These results are in good agreement with the XRD pattern (Figure 1b). The formation of the 2D phase and its crystallization are found to be dependent on the organic cation employed. To facilitate a more comprehensive understanding, we integrated the peak area corresponding to the 3D, PbI_2_, *n* = 1, *n* = 2, and unreacted salt as a function of time (Figure 4d–f).

The presence of XRD peaks related to unreacted salt for CHEAI- and PEAI-based 2D/3D perovskites was observed from the early moments of the deposition of the organic cation. In the case of the CHEAI samples, the intensity of PbI_2_ diffraction decreases upon the deposition of the organic cation, coinciding with the emergence of the *n* = 1 phase diffraction before thermal annealing.^41,42,57,58^ This indicates that the organic cation preferentially reacts with the excess lead iodide in the film to form the 2D phase. It is important to note that the intensity of the peak related to the salt in the case of CHEAI increases upon annealing, which suggests an improvement in the crystallinity of the salt. In other words, the salt crystallizes from a solution (or amorphous state) as the material is thermal annealed. This hypothesis is supported by the flexible nature of the CHEA^+^ cation, which would first form a poorly crystalline structure after its deposition. The thermal energy supplied during annealing drives the salt to a more crystalline state, which is reflected in a more intense diffraction feature. In the case of PEAI, the formation of 2D phases was observed exclusively upon thermal annealing, which is consistent with previous reports.^25,59,60^ The absence of the *n* = 2 phase for the CHEAI- and PEAI-based perovskites in the GIWAXS can be attributed to the fact that their main diffractions fall below the diffraction range analyzed.^25,27,45^ Additionally, as the *n* = 2 phase for these materials was not present in the XRD and was only very weak in the PL spectra, it is possible to assume that their GIWAXS signal is also under the detection limit of the technique. Finally, for the PCPEAI-based 2D/3D perovskite, the formation of *n* = 1 and *n* = 2 phases was observed only during thermal annealing, with no residual salt. The intensity of both diffractions exhibited a slight decrease over time. This suggests that the low-dimensional perovskites based on PCPEAI may be less stable than those based on CHEAI and PEAI. This can be attributed to the nature of the cyclopropyl ring. The C–C–C bond angle of this ring is 60°, which deviates significantly from the ideal tetrahedral bond angle of 109.5° for quaternary carbons. Furthermore, the three-membered ring is the least stable of the cycloalkyls.^61^ For example, the C–C bond in the cyclopropyl can cleave in the presence of a Lewis acid, even at room temperature, to form a less strained linear chain.^62^ Consequently, the 2D/3D perovskite may degrade over time due to a ring-opening reaction of PCPEAI. The presence of iodide vacancies on the surface of the film, which possess a Lewis acid character,^5^ can facilitate this reaction.

Our results demonstrate that the formation of the low-dimensional perovskite phases is highly dependent on the nature of the organic cation. The more flexible and less sterically hindered CHEAI cation forms the 2D phase immediately upon deposition onto the 3D perovskite. The replacement of the cyclohexyl group with a phenyl group in PEAI results in an increase in the molecular stiffness, which delays the formation of the 2D phase. This is likely due to the lower mobility of PEAI compared to the CHEAI cation. In both cases, we observed the presence of unreacted salt molecules together with the *n* = 1 phase. However, the CHEAI-based sample shows XRD peaks that are more intense for the unreacted salt in comparison to PEAI, which can be related to the flexibility of CHEAI, which may facilitate its crystallization. In the case of PCPEAI, we observe a delay in the formation of both *n* = 1 and *n* = 2 2D perovskite phases. However, these phases seem to not be stable under thermal annealing, which is associated with the high molecular stiffness and steric hindrance on the ammonium group and the instability of the cyclopropyl group, which is likely to undergo ring-opening reactions.

### 3.4 Impact on Perovskite Solar Cell Performance

We have demonstrated how the stiffness of bulky organic cations affects the formation and crystallization of 2D/3D perovskites. Now, we will examine the impact of these heterointerfaces on the efficiency of PSC. Regular *nip-*type PSCs were assembled with the architecture glass/FTO/SnO_2_/3D perovskite/2D phase/Spiro-OMeTAD/Au. Further details about the fabrication and characterization of the devices, with an active area of 0.16 cm^2^, can be found in the SI. For the device assembly, a thermal annealing step was conducted at 100 °C for 10 min following the deposition of the organic cation on the perovskite film.

**Figure 5:**
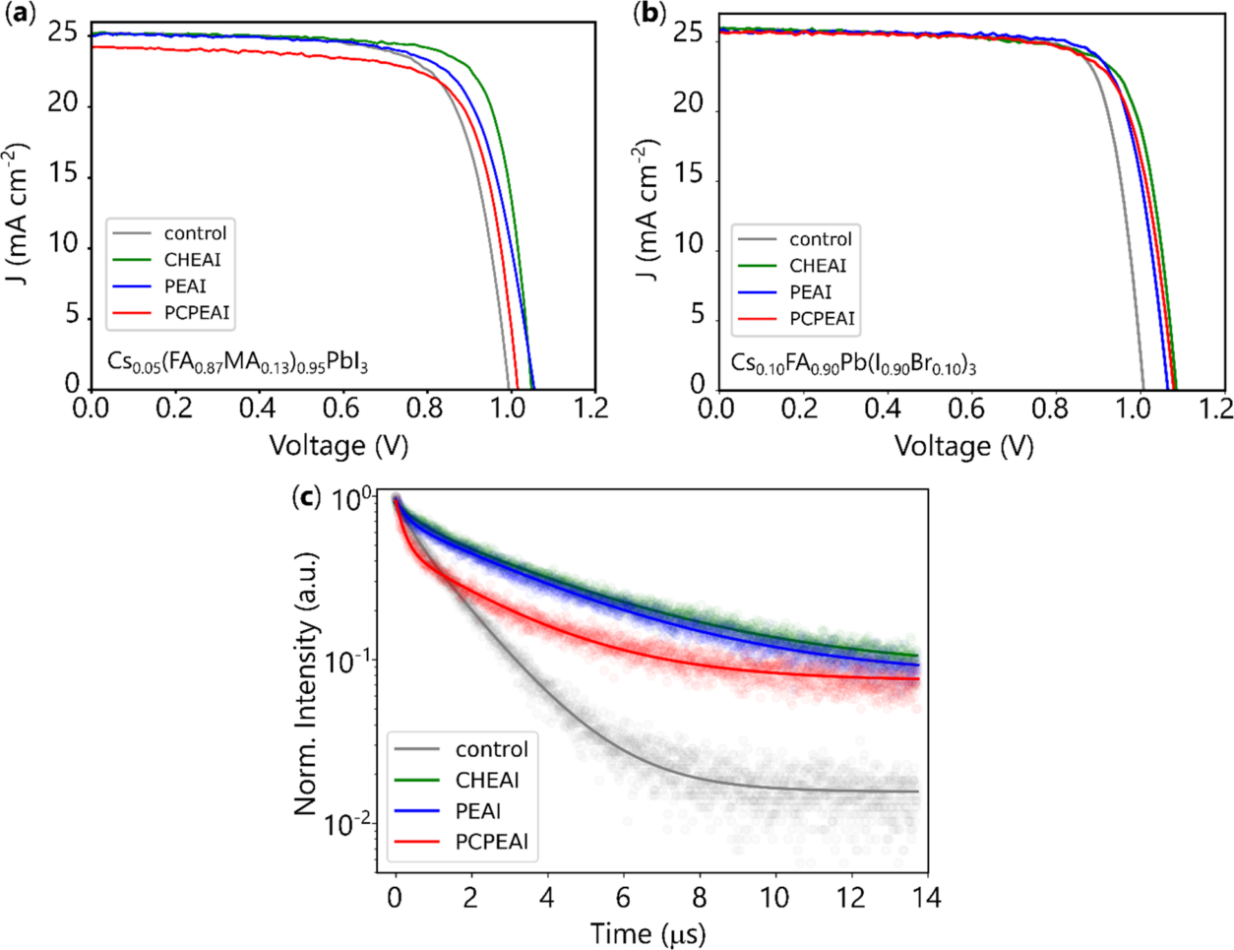
(a) Current density (*J*) versus voltage curve of the best-performing perovskite solar cells based on Cs_0.05_(FA_0.87_MA_0.13_)_0.95_PbI_3_ and (b) Cs_0.10_FA_0.90_Pb(I_0.90_Br_0.10_)_3_ as the active layer, and (c) time-resolved photoluminescence measurement of the control, CHEAI, PEAI, and PCPEAI-passivated films using Cs_0.05_(FA_0.87_MA_0.13_)_0.95_PbI_3_ as the active layer.

Two distinct perovskite compositions were employed: the aforementioned Cs_0.05_(FA_0.87_MA_0.13_)_0.95_PbI_3_ (CsFAMA) and another without MA and with the incorporation of bromide in the X-site, namely Cs_0.10_FA_0.90_Pb(I_0.90_Br_0.10_)_3_ (CsFA). It is worth mentioning that the other layers of the device were kept the same. Figure 5a,b presents the current density (*J*) versus voltage curves of the best-performing devices for the CsFAMA and CsFA compositions, respectively. The champion control device exhibits a maximum PCE of 18.17 and 20.66% for the CsFAMA- and CsFA-based PSCs, respectively. Tables S1 and S2 present the solar cell parameters for each composition. The most significant improvement obtained upon the bromide addition (associated with MA removal) is in the open-circuit voltage (*V*_OC_), which increased from an average of 0.97 V for the CsFAMA to 1.05 V for the CsFA. This improvement can be attributed to the band gap opening that occurs because of the bromide addition.^34,63,64^

The surface treatment using CHEAI, PEAI, and PCPEAI increases the device performance for both perovskite compositions. As shown in Figures S9 and S10, the main improvements are in *V*_OC_, followed by the fill factor (FF). For the CsFAMA-based PSC, the average efficiency increased from 16.6% for the control to 18.8, 17.9, and 17.3% for CHEAI, PEAI, and PCPEAI, respectively. The best-performing device was obtained with CHEAI passivation, with a PCE of 20.47%. For the CsFA-based devices, the average efficiency increased from 19.4% for the control to 20.3, 19.7, and 18.8% after surface passivation with CHEAI, PEAI, and PCPEAI, respectively. For this perovskite composition, CHEAI and PEAI presented record efficiencies of 21.5% each. These results demonstrate that the surface passivation strategy is valid for different perovskite compositions.

In general, there is an increase in the *V*_OC_ of the 2D/3D perovskites compared to the pristine 3D film (Figures 5a,b). This suggests that the formation of the 2D phase at the grain boundaries of the 3D perovskite, as shown from the SEM-CL analysis, reduces the nonradiative recombination rate. To test this hypothesis, we used time-resolved photoluminescence (TRPL) spectroscopy on CsFAMA/2D heterointerface films to measure the average carrier lifetime (Figure 5c). The decays were fitted with a double exponential equation, resulting in two different time constants. After passivation, the average lifetime increased for all organic cations tested, going from 1.20 μs for the control to 3.20, 2.75, and 1.35 μs for CHEAI, PEAI, and PCPEAI, respectively (Table S3). The higher average lifetime obtained for the passivated films follows what was observed in the *V*_OC_ and indicates that the organic cations passivate trap states on the film.^39,65^ Since the *V*_OC_ improvements were also observed for the CsFA-based devices, it is reasonable to assume that the carrier lifetime also increased after the passivation of these films with the organic cations. We notice that passivation with CHEAI and PEAI results in a similar passivation effect. Conversely, passivation with PCPEAI has only a slight effect on *V*_OC_. This can be attributed to the strained conformation of the cyclopropane ring of this molecule, which may prevent PCPEAI from interacting with defects present on the 3D film surface, or due to the degradation of the PCPEAI-based 2D phase.

Next, we investigate the recombination mechanism across the CsFAMA/2D heterointerface using Electrochemical Impedance Spectroscopy (EIS). The Nyquist plots show two arcs (Figure S11): the first at high frequency (HF), commonly associated with the charge transfer process, and the second at low frequency (LF). The LF arc has been extensively discussed in the literature, with some consensus regarding contributions from recombination effects.^28,66−68^ The fitted parameters are listed in Table S4.

The results indicate that the formation of the 2D phase on the perovskite generally increases the charge transfer resistance (*R*_ct_). This is probably due to the high concentration of organic cations used, as mentioned above. These molecules have a low dielectric constant that can prevent charge transfer from the perovskite to the hole transporting material. In addition, it is well-known that low-dimensional RP perovskites have unmatched energy levels compared to bulk films, which may hinder (at least partially) the current flow from the perovskite to the hole transport material.^69−71^ However, it is worth noting that CHEAI has a much lower *R*_ct_ than PEAI while still maintaining a high value of *R*_LF_ (which describes the recombination resistance, *R*_rec_). The lower *R*_ct_ for the CHEAI-based 2D/3D perovskite can be attributed to the smoother perovskite surface obtained after the formation of the 2D phase (see Figure S2). This helps explain why CHEAI-treated devices perform better (in average) than others. CHEAI can passivate defects, which increases *R*_rec_ and promotes a better charge flow across the perovskite/HTL interface. On the other hand, PCPEAI has the lowest *R*_ct_. Interestingly, the trend in *R*_ct_ matches the trend in molecular flexibility, i.e., CHEAI > PEAI > PCPEAI. The increased flexibility in CHEAI allows the molecule a higher degree of freedom to adopt different conformations and contributes to a smoother interface.^41^ Nevertheless, for PCPEAI, the lower *R*_rec_, which implies high carrier recombination, prevents PCE from being as high as CHEAI.

## 4 Conclusions

The formation and temperature-dependent crystallization of 2D/3D perovskite heterointerfaces prepared from ammonium-based organic cations with different degrees of stiffness have been demonstrated. First, the distribution of the 2D phases on the film was studied from the micro- to the nanoscale. Using SEM-CL, we demonstrated that the 2D perovskite phases are preferentially formed at the grain boundaries of the 3D perovskite with an inhomogeneous distribution of the different *n*-values 2D phases. Next, we investigated the formation and crystallization of the 2D phase on the 3D perovskite and its dependence on the stiffness of the organic cation. Among the cations used, CHEAI and PEAI have identical alkylammonium chains, differing in the presence of a cyclohexyl ring instead of a phenyl ring in CHEAI. PCPEAI has a phenyl ring like PEAI but a cycloalkylammonium instead of a linear alkylammonium chain. In this series of molecules, we observed that the formation and crystallization of the 2D phase on the 3D phase are closely related to the molecular stiffness and steric hindrance on the ammonium group. Finally, we evaluated the performance of 2D/3D perovskite interfaces in solar cells. We found that the more flexible and sterically available CHEAI cation resulted in a higher performance of perovskite solar cells. The formation of the low-dimensional phases at the grain boundaries helps explain the improvement observed, as grain boundaries are defect sites in the perovskite. The results highlight the intricate relationship between the formation of 2D perovskites and the flexibility of the aryl/alkyl group in bulky organic ammonium-based cations.

## Supporting Information Available

Device assembly procedure; details about the characterization techniques; additional figures regarding XRD, SEM, and CL; statistics of the *V*_OC_, *J*_SC_, FF, and PCE of the devices; EIS Nyquist spectra (PDF)
