# Supplementary material for: 2D Phase Formation on 3D Perovskite: Insights from Molecular Stiffness
Source: ACS Appl Mater Interfaces. 2024 Sep 13;16(38):51727–37. doi: 10.1021/acsami.4c11394 (PMC11440457; doi:10.1021/acsami.4c11394)
Supplement: Supplementary file 1 — am4c11394_si_001.pdf [file am4c11394_si_001.pdf]

Supporting Information

2D Phase Formation on 3D perovskite: Insights from Molecular Stiffness

Lucas Scalon[a‡], Charles Alves Nogueira[b‡], André Felipe Vale Fonseca[a], Paulo E. Marchezi[ac], Raphael Fernando. Moral[ad], Giulia Grancini[e], Tim Kodalle[df], Carolin M. Sutter-Fella[d], Caio Costa Oliveira[a], Luiz F. Zagonel[b], Ana F. Nogueira[a*]*

[a] *Institute of Chemistry, University of Campinas (UNICAMP), 13083-970 Campinas, São Paulo, Brazil.*

[b] *Gleb Wataghin Institute of Physics, University of Campinas (UNICAMP), 13083-859 Campinas, São Paulo, Brazil.*

[c] *Department of Nanoengineering, UC San Diego, 9500 Gilman Drive, La Jolla, 92093 California, United States.*

[d] *Molecular Foundry, Lawrence Berkeley National Laboratory, 1 Cyclotron Road, Berkeley 94720 California, United States.*

[e] *Department of Chemistry and INSTM, University of Pavia, Via T. Taramelly 14, 27100 Pavia, Italy.*

[f] *Advanced Light Source, Lawrence Berkeley National Laboratory, 1 Cyclotron Road, Berkeley 94720 California, United States.*

[*] Email: zagonel@unicamp.br; anafla@unicamp.br

[‡] L.S. and C.A.N. contributed equally to this paper

Materials and methods

Solar cell assembly: glass/FTO substrates were cleaned with Hellmanex solution 2% in deionized (DI) water, acetone, and isopropanol in an ultrasound bath for 20 min each. Then, the substrates were dried with nitrogen, and treated in a UV-ozone chamber for 30 min. The SnO_2 layer was deposited by diluting a 15 wt% stock solution in DI water in a 1:4 proportion and adding 3 mg mL^{-1} of KCl to passivate the SnO_2 layer and decrease hysteresis. This solution was spin-coated onto the FTO substrate at 4000 rpm, for 30 s, with an acceleration of 2,000 rpm s^{-1}, followed by a thermal annealing at 150 °C for 30 min. The films were then submitted to a surface treatment in a UV-ozone chamber for 30 min and immediately transferred to a nitrogen-filled glove box for the perovskite deposition. Then, the perovskite was deposited according to the previous protocol. After that, 55 µL of the organic cation solutions in isopropanol (40 mM) were dynamically deposited onto the perovskite surface at 4,000 rpm, for 30 s, followed by thermal annealing at 100 °C for 10 min. Finally, a solution containing 97 mg of Spiro-OMeTAD, 42.4 µL of 4- tert-butyl pyridine, and 24.8 µL of Li-TFSI solution (520 mg mL^{-1} in ACN), and 10.8 µL of FK209 (375 mg mL^{-1} in ACN) in 1 mL chlorobenzene, was deposited on perovskite films at 4,000 rpm for 25 s. For the Spiro-OMeTAD oxidation, the FTO/SnO_2/perovskite/Spiro-OMeTAD films were kept in a dissector with silica, protected from the light, in an environment with humidity < 35%, for at least 12 h. To complete the device fabrication, 70 nm of Au was thermally evaporated using a rate of 0.1 Å s^{-1} (4 nm) and 1.0 Å s^{-1} (66 nm).

Device characterization: the current density v*s.* voltage (J–V) curves were characterized under simulated air mass (AM) 1.5 G illumination using a solar simulator (450 W xenon light source (Oriel), 100 mW cm^{-2}) and a Keithley 2400 source meter. The light intensity was calibrated with a Si photodiode equipped with an IR-cutoff filter (KG3, Schott). The solar cells were measured under reverse scan (+1.2 V to 0 V) and forward scan (0 V to +1.2 V) with a scan rate of 50 mV s^{-1}. A shadow mask of 0.16 cm^2 was used to delimit the active area of the device and avoid overestimation of short-circuit current.

Impedance Spectroscopy (IS): IS was registered using a Metrohm Autolab PGSTAT204, with a sinusoidal perturbation of 100 mV, with frequencies ranging from 100 mHz to 1 MHz in a logarithmic distribution in 60 points, and at 0.4 V bias. The IS was measured under simulated air mass (AM) 1.5 G illumination using a solar simulator (450 W xenon

light source (Oriel) 30 mW cm^{-2}). It employed a 0.3-sun power to prevent light exposure damage, as recently recommended in the literature.[1]

Scanning Electron Microscopy: the morphology characterizations were collected using a Helios 5 PFIB CXe DualBeam at the Microscopic Samples Laboratory (LAM-FIB), at CNPEM. The microscope was adjusted to low electron beam acceleration voltage, 5 kV or 2 kV. To reduce charging and damage to the samples, we covered the sample surface with 60 nm thick amorphous carbon using Cressington 108 series automatic SEM carbon coater.

Cathodoluminescence: CL measurements were carried out at the Microscopic Samples Laboratory (LAM-FIB), at CNPEM. The equipment used was installed in the same SEM used for morphology characterization. The CL system is a SPARC Spectral CL detector by Delmic equipped with a Kymera 193i spectrograph, and a CCD Newton DU920P-BEX2-DD-E23 from Andor.

X-Ray Diffraction: the XRD patterns of the 2D/3D perovskite films after thermal annealing at 100 °C for 10 min were taken with a Shimadzu XRD-6000 X-ray diffractometer using Cu Kα radiation of 8 keV and an incident angle of 1.39°. The films were prepared onto glass substrates.

Steady-state photoluminescence: the emission signal of the 2D/3D perovskite films after thermal annealing at 100 °C for 10 min was recorded by an Ocean Optics QE-Pro spectrofluorometer through an optical fiber. The samples were excited by a 365 nm LED (Ocean Optics, model) coupled to another optical fiber. A 400 nm filter (Thorlabs) was employed before the emission-collector optical fiber to prevent excitation scattering effects on the emission signal. The films were prepared onto a glass substrate.

Time-resolved photoluminescence: measurements of the 2D/3D perovskite films after thermal annealing at 100 °C for 10 min were performed in a spectrofluorometer (Horiba, Nanolog) equipped with a photon-counting detector (PPD850, Horiba) and a module of time-correlated single photon counting (TCSPC). The sample was excited with a 500 nm laser. The films were prepared onto a glass substrate.

In situ GIWAXS: the measurements were performed in the 12.3.2 beamline at the Advanced Light Source Synchrotron, Lawrence Berkeley National Laboratory in Berkeley, California, USA. A home-made remotely powered heated spin-coater was

installed in the experimental beamline hutch for in situ measurements in nitrogen atmosphere. The X-ray energy was 10 keV, with an incident angle of 1° using the Dectris Pilatus 1M detector at a distance of approximately 160 mm from the sample and with an inclination of 35° in relation to the direct beam. Calibration was performed using ITO standard. An integration of 2.35 s was used; the readout time was 0.66 s per acquisition.

Supplementary Figures and Tables

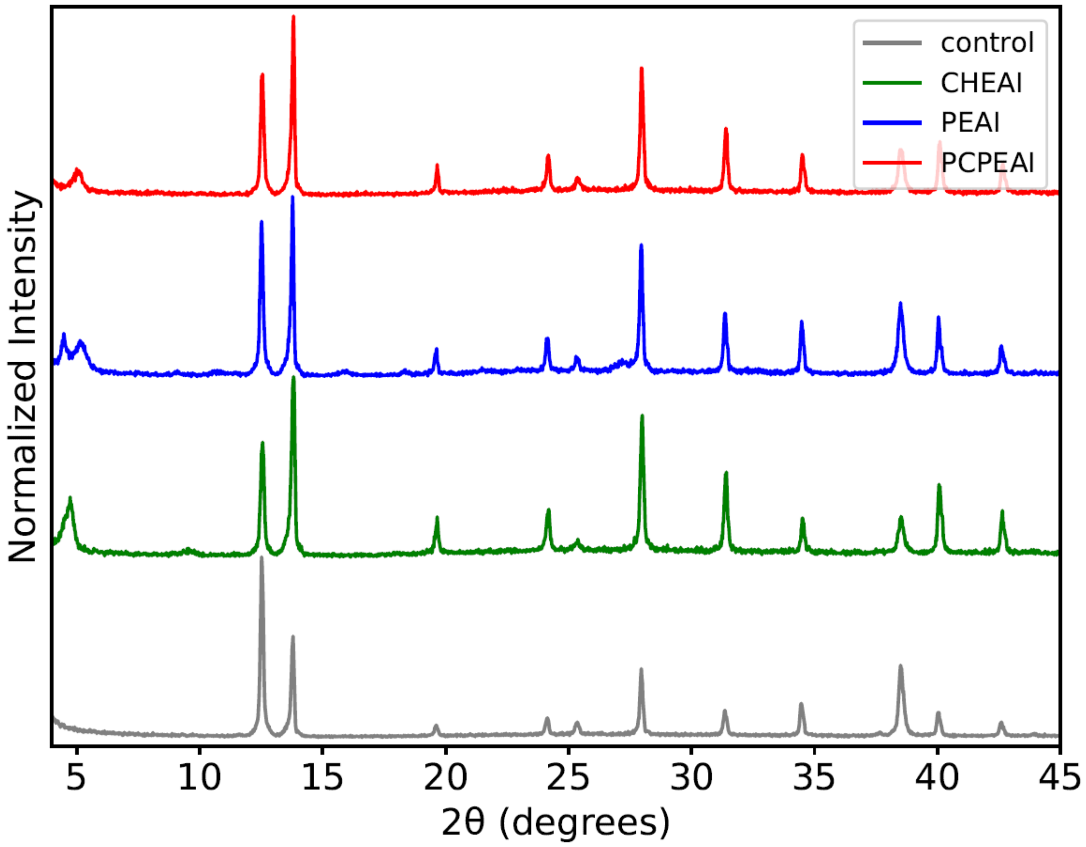

Figure S1. XRD of the 3D and CHEAI-, PEAI-, PCPEAI-based 2D/3D perovskite films. X-ray energy: 8 keV. Incident angle: 1.39°.

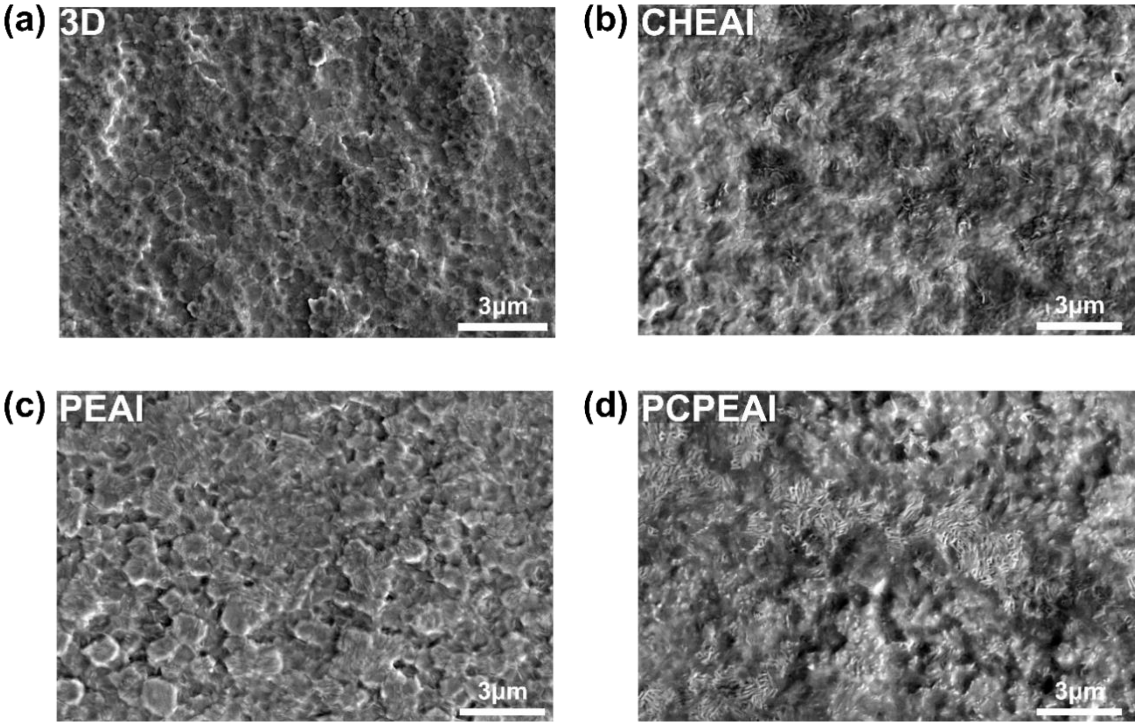

Figure S2. SEM images of the (a) pristine 3D perovskite acquired using 5 kV and 0.10 nA, and the 2D/3D perovskites prepared from (b) CHEAI, (c) PEAI, and (d) PCPEAI, acquired using 2 kV and 0.20 nA, 5 kV and 0.10 nA, and 2 kV and 0.20 nA, respectively.

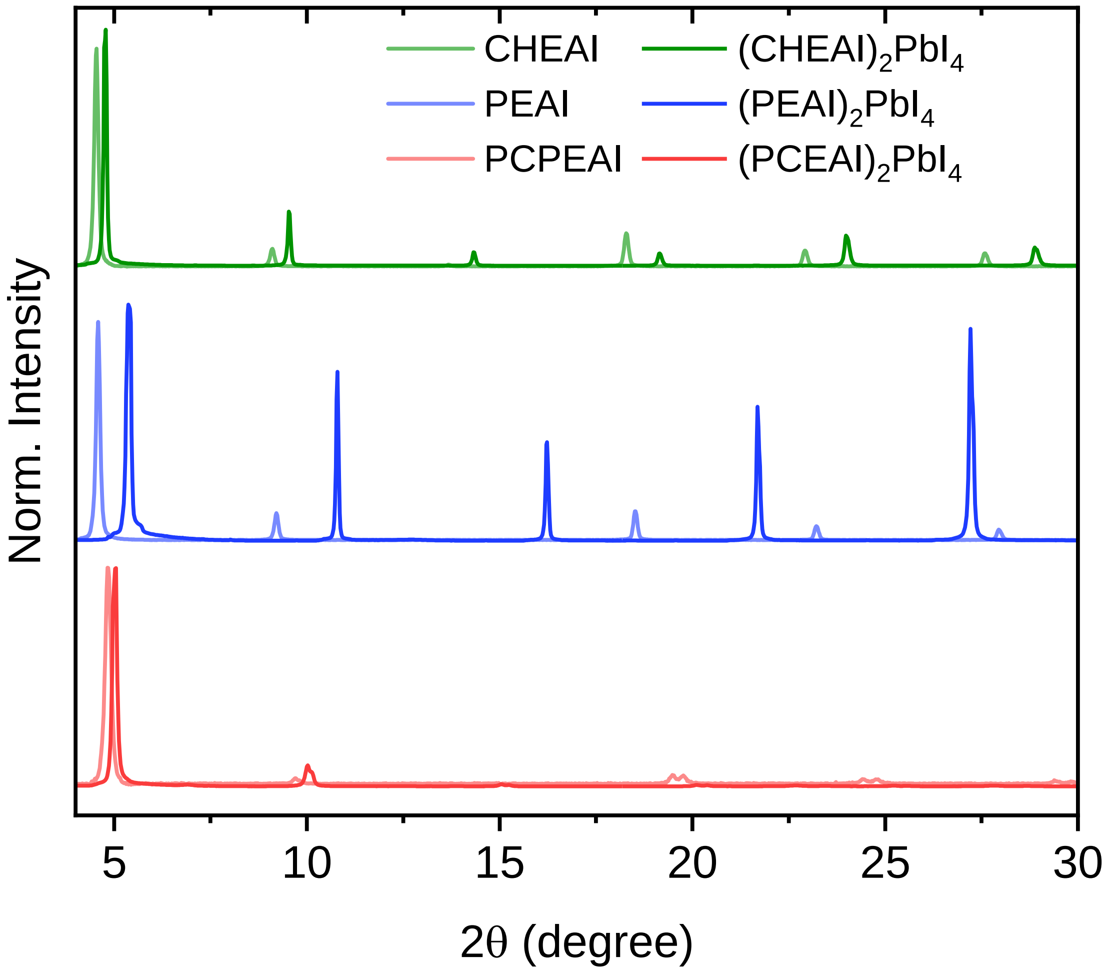

Figure S3. XRD of the CHEAI, PEAI, and PCPEAI salts (light lines) and the corresponding 2D n=1 perovskite prepared from these cations (bold lines). X-ray energy: 8 keV. Incident angle: 1.39°.

Support Information Note 1

The cathodoluminescence technique

Unlike conventional SSPL measurements that use light (*e.g.*, a laser) as the excitation source, SEM-CL employs an electron beam (*e*-beam) to excite the sample. Because of the smaller wavelength of the electrons in SEMs, the Abbe diffraction limit, which is approximately λ/2NA, where λ is the wavelength and NA is the numerical aperture of the microscope imaging system, is reduced.[2] As a result, SEM-CL permits obtaining a better spatial resolution (~20 nm) while locally exciting luminesce, which allows the 3D and 2D phase emissions to be spatially and spectrally distinguished. Additionally, it is possible to correlate the local luminescence in the sample with its morphology and microstructure at the nanoscale.[3]

The SEM-CL has two operation modes: spectrum imaging and energy-filtered imaging (**Figure S4**). The main difference is the detector used: while in spectrum imaging the emitted light is recorded by a CCD (Charge-Coupled Device) camera (a multichannel detector) inside a spectrometer (which disperses the light according to wavelength), in energy-filtered mode the light is recorded by a PMT (Photomultiplier tube, which is a single channel detector) after a band-pass filter. This means that, in the first mode, a full spectrum for each electron beam position is obtained, and, in the second mode, only the total light intensity for an energy (wavelength) range is obtained. The spectral range in the CCD can be adjusted using the spectrometer diffraction gratings and the spectral range in the PMT can be adjusted using optical filters. Spectral images are a 3D data set (a 1D array of intensity for each wavelength, that is, a spectrum, for each 2D position on the sample surface, that is an image) while energy-filtered images are a 2D data sets, that is a light-intensity image. Spectral images contain much more information and can be displayed in a number of ways (including as light-intensity images in arbitrary energy range) but require much more exposure to the electron beam while energy-filtered images are very fast to acquire. Energy-filtered images can also be acquired without the optical filter in which case a broader energy range is recorded, and the image is called panchromatic image.

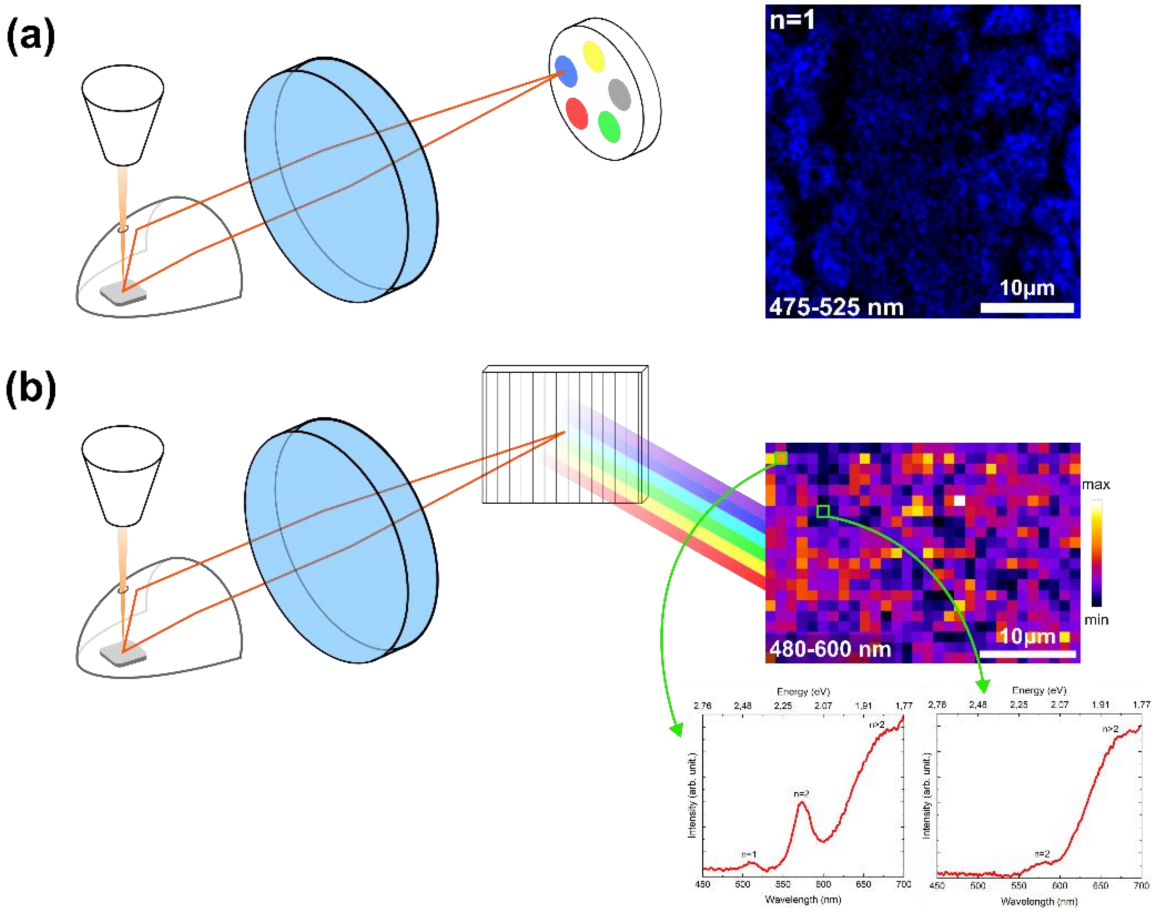

Figure S4. A simplified schematic of the SEM-CL technique. The luminescence emitted by the sample (when excited by the electron beam) is reflected by the parabolic mirror and passed through a series of lenses. The light can be collected in two modes: (a) by passing through a band-pass filter of a wavelength chosen by the operator and then recorded by a PMT, or (b) by passing through the spectrometer, where it is dispersed in wavelength by a diffraction grating and then recorded by a CCD. The second mode results in a 3D data set (1D array of intensity for each wavelength for each 2D position on the sample surface). That is: each position in the spectrum imaging records a CL spectrum.

Support Information Note 2

Controlling the accelerating voltage

By controlling the accelerating voltage, we are able to extract information from different depths of the film (**Figure S5**). For example, an accelerating voltage of 2 kV allows tracking only the topmost layer, composed mainly of 2D perovskite domains. However, increasing the accelerating voltage to 5 kV or 10 kV reveals the 3D perovskite grains. This approach facilitates the monitoring of the 2D phase and the perovskite film independently.

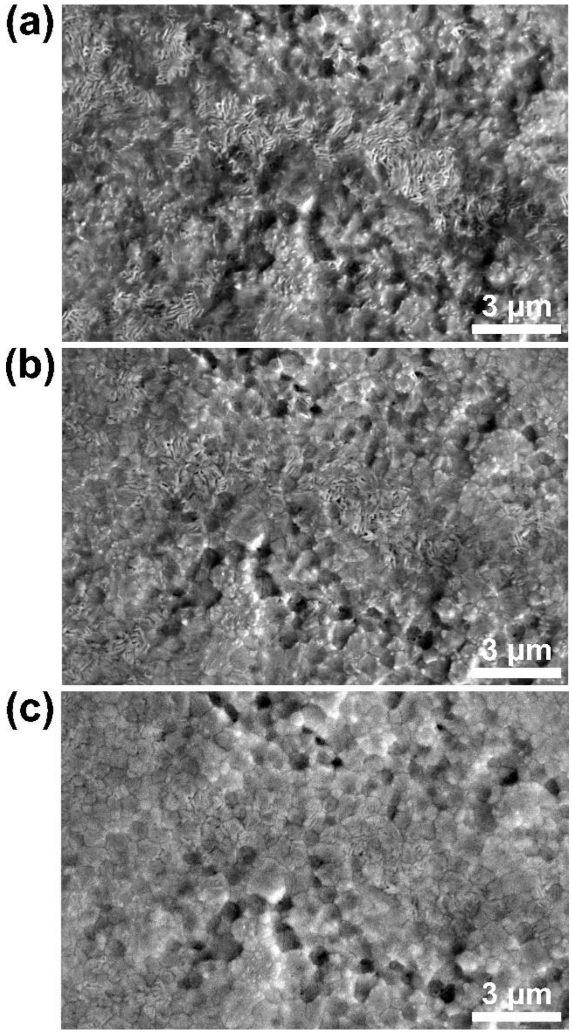

Figure S5. SEM images of the PCPEAI sample surface, acquired at the same are increasing the accelerating voltage by (a) 2 kV, (b) 5 kV, and (c) 10 kV.

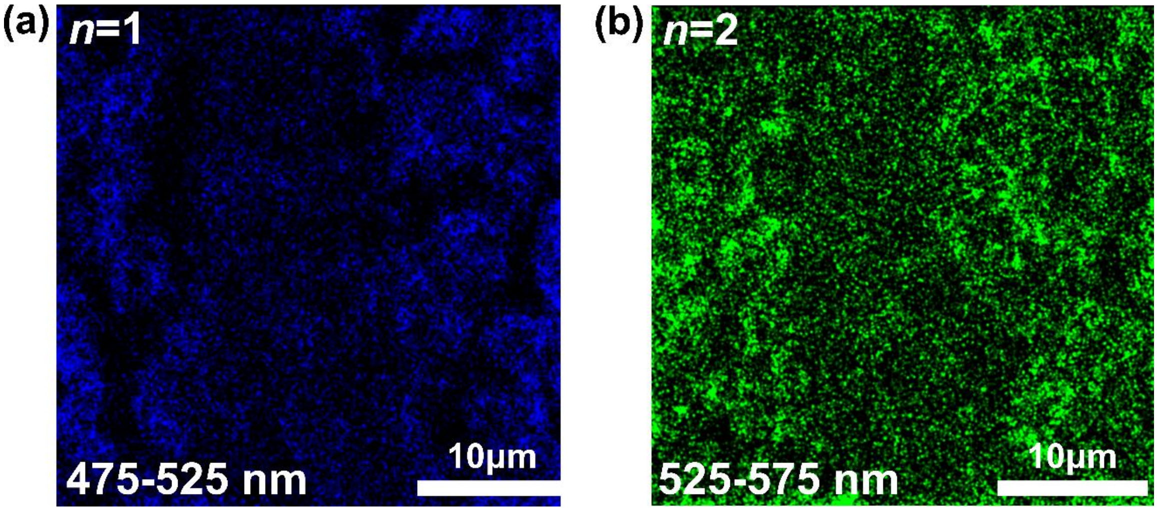

Figure S6. CL panchromatic image with a filter in the ranges from 475 to 525 nm in (a), and the range of 525 to 575 nm in (b), acquired simultaneously with the SEM image in **Figure 2d**.

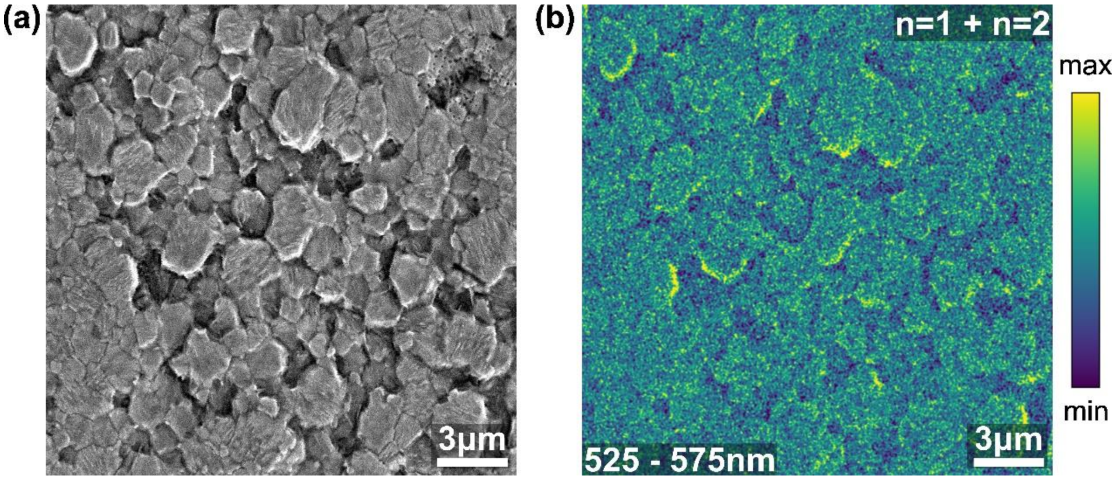

Figure S7. (a) SEM image of the PEAI-based 2D/3D perovskite film and (b) CL panchromatic image with a filter in the ranges from 525 to 575 nm.

Support Information Note 3

CL-SEM PCPEAI-based 2D/3D perovskite

Figures S8a and **b** illustrate the SEM image and the corresponding panchromatic CL image. As can be seen in **Figure S8b**, there is an uneven distribution of emissions across the film. The large-area CL spectrum (**Figure S8e**) shows that the emission is mostly due to the 3D phase, which is consistent with the SSPL spectra (**Figure 1d**). Upon setting a filter at the *n*=2 phase emission (**Figure S8d**), the non-homogeneous distribution of the 2D phase becomes evident, indicating that the *n*=2 phase is located at the needle-like domains.

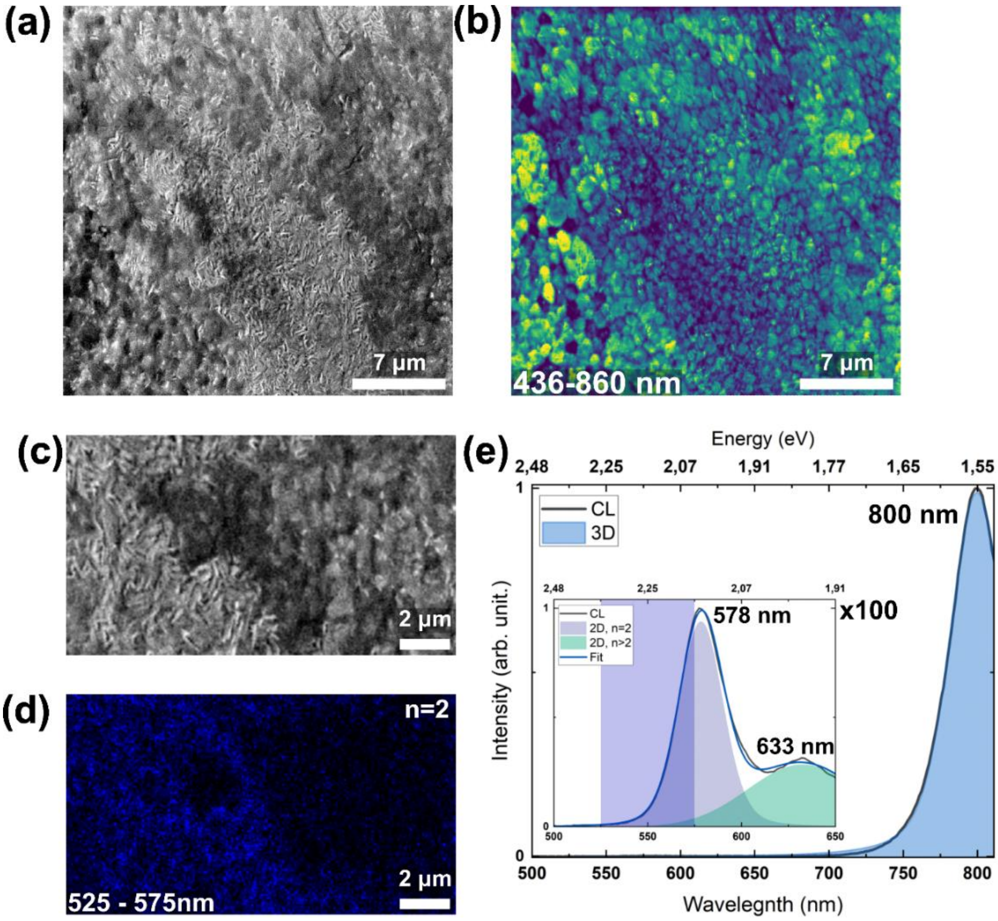

Figure S8. (a) SEM image of the PCPEAI-based 2D/3D perovskite film and (b) CL panchromatic image of the same area as (a). (c) SEM showing the needle-like grains and (d) CL image with a filter in the ranges from 525 nm to 575 nm of the same area as (c). (e) Large area CL spectrum collected in the region of 500-860 nm. The inset shows the same spectrum in the range of the 2D phases. The wavelengths of the peaks are indicated. The range highlighted in blue in the spectrum (inset) is associated with the 2D phase, *n*=2. The CL spectrum was acquired with 2 kV and 200 pA.

Table S1. Average, standard deviation and maximum efficiency of the perovskite solar cells based on $Cs_{0.05}(FA_{0.87}MA_{0.13})_{0.95}PbI_3$ as an active layer.

Device name	Direction	V_{OC} (V)	J_{SC} (mA cm^{-2})	*FF* (%)	PCE (%)
**Control**	B	0.97±0.02	25.09±0.17	68.51±3.78	16.60±0.99
	F	0.92±0.03	25.10±0.02	60.81±3.21	14.02±1.03
	**MAX.**	**0.99**	**25.21**	**72.81**	**18.17**
**CHEAI**	B	1.03±0.02	24.78±0.24	73.94±2.59	18.84±1.04
	F	0.98±0.05	24.80±0.30	66.88±4.46	16.30±1.89
	**MAX.**	**25.13**	**1.05**	**77.58**	**20.47**
**PEAI**	B	1.04±0.02	24.67±0.58	69.99±1.23	17.93±0.79
	F	0.99±0.04	24.68±0.64	63.73±3.12	15.63±1.56
	**MAX.**	**25.04**	**1.05**	**72.57**	**19.08**
**PCPEAI**	B	1.02±0.02	24.51±0.55	69.35±4.72	17.31±1.20
	F	0.99±0.02	24.79±0.54	63.69±3.31	15.58±0.86
	**MAX.**	**1.02**	**24.19**	**74.38**	**18.35**

Table S2. Average, standard deviation and maximum efficiency of the perovskite solar cells based on $Cs_{0.10}FA_{0.90}Pb(I_{0.90}Br_{0.10})_3$ as an active layer.

Device name	Direction	V_{OC} (V)	J_{SC} (mA cm^{-2})	*FF* (%)	PCE (%)
**Control**	B	1.05±0.02	25.13±0.39	73.37±3.86	19.42±1.03
	F	1.03±0.02	25.10±0.43	69.58±3.65	18.01±0.95
	**MAX.**	**1.01**	**25.88**	**79.05**	**20.66**
**CHEAI**	B	1.09±0.02	25.19±0.59	74.26±3.70	20.30±1.06
	F	1.07±0.03	25.21±0.58	72.57±2.69	19.52±0.98
	**MAX.**	**1.08**	**25.93**	**76.73**	**21.50**
**PEAI**	B	1.08±0.02	25.11±0.73	73.03±4.65	19.70±1.15
	F	1.05±0.03	25.09±0.74	69.69±4.26	18.48±1.43
	**MAX.**	**1.06**	**25.7**	**78.94**	**21.50**
**PCPEAI**	B	1.03±0.03	25.26±0.45	72.18±5.98	18.75±1.59
	F	1.01±0.03	25.20±0.43	70.53±3.38	17.99±0.96
	**MAX.**	**1.08**	**25.69**	**75.58**	**20.97**

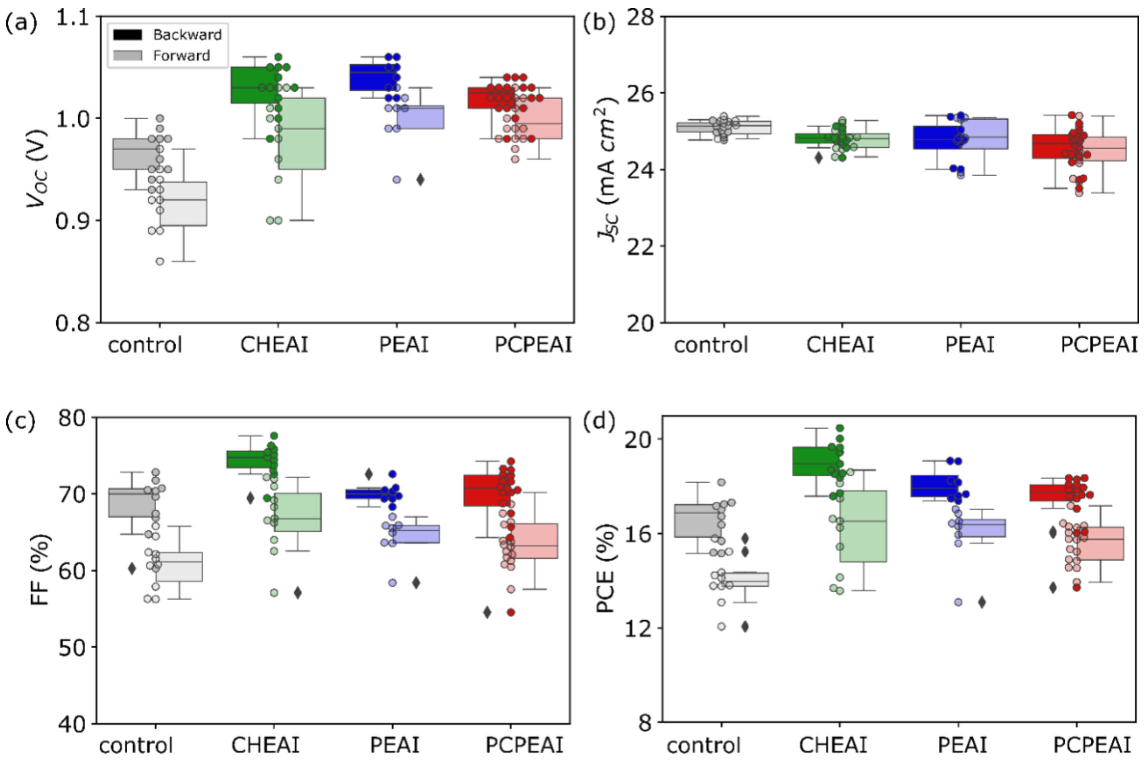

Figure S9. Statistics of (a) open-circuit voltage (V_{OC}), (b) short-circuit current (J_{SC}), (c) fill factor (FF), and (d) power conversion efficiency (PCE) of perovskite solar cells based on $Cs_{0.05}(FA_{0.87}MA_{0.13})_{0.95}PbI_3$ as the active layer.

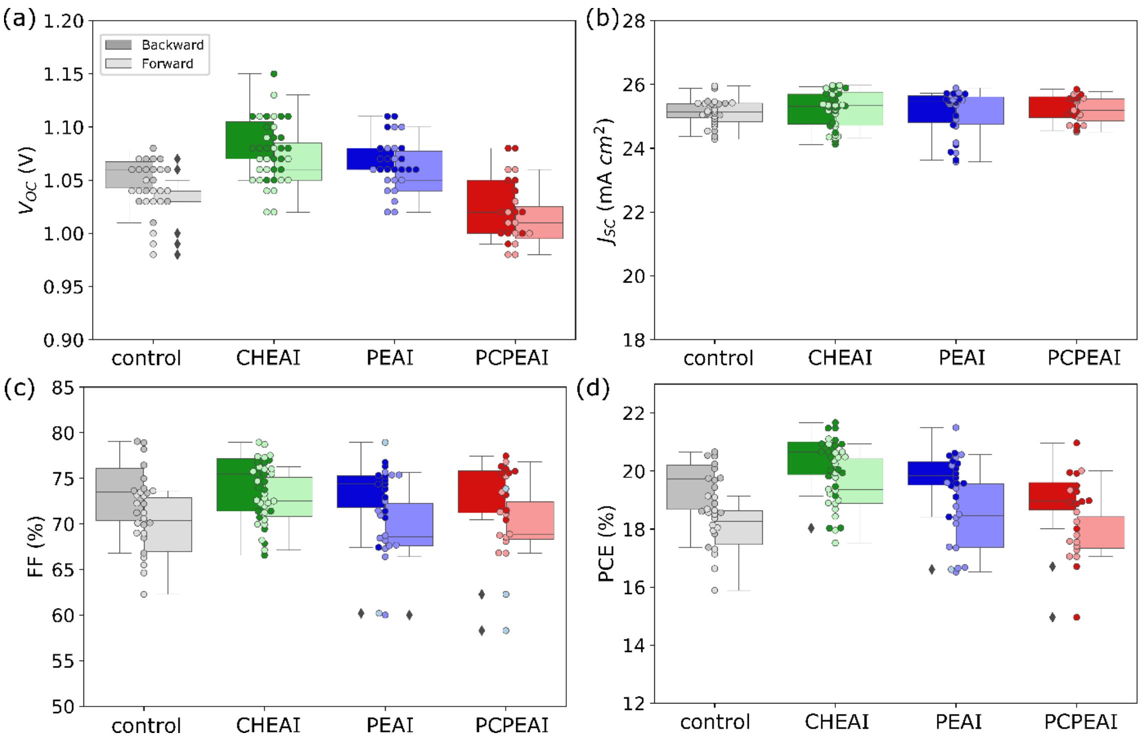

Figure S10. Statistics of (a) open-circuit voltage (V_{OC}), (b) short-circuit current (J_{SC}), (c) fill factor (FF), and (d) power conversion efficiency (PCE) of perovskite solar cells based on $Cs_{0.10}FA_{0.90}Pb(I_{0.90}Br_{0.10})_3$ as the active layer.

Table S3. Lifetime fitting on decays in **Figure 6** using the double-exponential modeling.

**Sample**	**A_1**	**τ_1 (µs)**	**Integrated contribution**	**A_2**	**τ_2 (µs)**	**Integrated contribution**	**τ_{avg}* (µs)**
**Control**	0.229	0.336	6.8	0.713	1.47	93.2	1.20
**CHEAI**	0.184	0.617	4.3	0.631	3.958	95.7	3.20
**PEAI**	0.236	0.289	2.9	0.631	3.673	97.1	2.75
**PCPEAI**	0.449	0.220	8.6	0.406	2.596	91.4	1.35

*: The average lifetime was calculated using the Equation 1:

$$t_{avg} = \frac{\sum a_i \tau_i}{\sum a_i} \qquad \textbf{Equation 1}$$

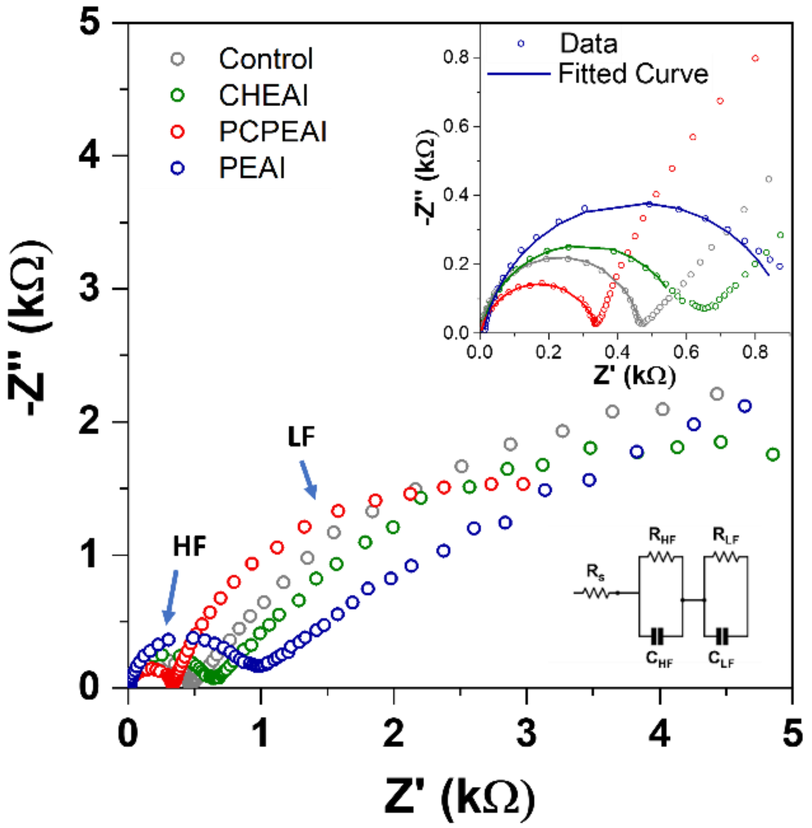

Figure S11. EIS Nyquist spectra measured under AM 1.5 (0.3 sun) illumination and at 0.4 V applied potential. Inset: The high-frequency region highlighted, and the fitted equivalent circuit.

Table S4. Equivalent circuit parameters fitted from EIS data.

Sample	R_s (Ω)	R_{HF} (Ω)	C_{HF} (x10^{-8} F)	R_{LF} (kΩ)	C_{LF} (x10^{-4} F)
**Control**	5.13	459.27	1.44	8.82	1.94
**CHEAI**	4.09	583.47	2.55	7.12	1.11
**PEAI**	11.08	899.88	2.18	14.22	1.18
**PCPEAI**	4.19	336.21	2.03	4.29	2.18

References

1. Pitarch-Tena, D., Ngo, T.T., Vallés-Pelarda, M., Pauporté, T., and Mora-Seró, I. (2018). Impedance spectroscopy measurements in perovskite solar cells: device stability and noise reduction. ACS Energy Lett. *3*, 1044–1048. 10.1021/acsenergylett.8b00465.

2. McLeod, E., and Ozcan, A. (2014). Nano-imaging enabled via self-assembly. Nano Today *9*, 560–573. 10.1016/j.nantod.2014.08.005.

3. Jose Chirayil, C., Abraham, J., Kumar Mishra, R., George, S.C., and Thomas, S. (2017). Instrumental techniques for the characterization of nanoparticles. In Thermal and rheological measurement techniques for nanomaterials characterization (Elsevier), pp. 1–36. 10.1016/B978-0-323-46139-9.00001-3.
